# PURE MATURE TERATOMA IN POST-PUBERTAL TRANSGENDER FEMALE

**DOI:** 10.51894/001c.123010

**Published:** 2024-08-30

**Authors:** Mercedes Kent

**Affiliations:** 1 Urology Sparrow Hospital

## 51

### INTRODUCTION

Approximately 8,000 men are diagnosed with testicular cancer annually. Though physical exam, laboratory values and imaging findings can be suggestive of malignancy, testicular cancer is ultimately diagnosed with pathology specimen after orchiectomy. Pathologically, testicular cancer is divided into germ cell tumors (GCTs) and non-germ cell tumors (non-GCTs). GCTs are further divided into seminoma and non-seminoma. The later includes teratoma, embryonal carcinoma, choriocarcinoma and yolk sac tumors. Teratomas usually exist as a component of a mixed germ cell tumor-including components of seminoma and/or non-seminoma. A pure teratoma -meaning no other testicular tumor components- tends to be associated with a pediatric, pre-pubertal patient. Additionally, there is an association risk factor attributed to in utero estrogen exposure and later development of testicular cancer.

### CASE DESCRIPTION

Patient WN is a 20-transgender female who noticed right testicular swelling noted to be suspicious for malignancy on imaging. A repeat ultrasound approximately one year later again demonstrated findings suspicious for mass, but it had grown in size. They were able to establish care with a urologist who discussed recommended right sided orchiectomy, performed 09/2023. Pathology demonstrated pure mature teratoma.

### DISCUSSION

As discussed above, this finding is worth noting given their post-pubertal status in addition to lack of other germ cell components. Furthermore, their history of hormone therapy including estradiol and spironolactone may play a role in the development of testicular cancer similarly to the established relationship of estrogen in utero. Therefore, it is worth examining supplemental hormone use and the prevalence and/or associated pathology and prevalence of testicular cancer, particularly as cases such as this may become more prevalent in time.

